# Implementing and Sustaining Early Cancer Diagnosis Initiatives in Canada: An Exploratory Qualitative Study

**DOI:** 10.3390/curroncol28060369

**Published:** 2021-10-30

**Authors:** Christine Fahim, Larkin Davenport Huyer, Tom (Taehoon) Lee, Anubha Prashad, Robyn Leonard, Satya Rashi Khare, Jennifer Stiff, Jennifer Chadder, Sharon E. Straus

**Affiliations:** 1 Knowledge Translation Program, Li Ka Shing Knowledge Institute, St. Michael’s Hospital, Unity Health, Toronto, ON M5B 1W8, Canada; larkin.davenporthuyer@unityhealth.to (L.D.H.); tom.lee@unityhealth.to (T.L.); sharon.straus@unityhealth.to (S.E.S.); 2 Canadian Partnership Against Cancer, Toronto, ON M5H 1J8, Canada; Anubha.Prashad@partnershipagainstcancer.ca (A.P.); Robyn.Leonard@partnershipagainstcancer.ca (R.L.); rashi.khare@partnershipagainstcancer.ca (S.R.K.); jennifer.stiff@partnershipagainstcancer.ca (J.S.); Jennifer.Chadder@partnershipagainstcancer.ca (J.C.)

**Keywords:** cancer diagnosis, early cancer diagnosis initiatives, health services research, implementation, evaluation

## Abstract

Background: The interval between suspected cancer and diagnosis for symptomatic patients is often fragmented, leading to diagnosis delays and increased patient stress. We conducted an exploratory qualitative study to explore barriers and facilitators to implementing and sustaining current initiatives across Canada that optimize early cancer diagnosis, with particular relevance for symptomatic patients. Methods: The national study included a document review and key informant interviews with purposefully recruited participants. Data were analyzed by two researchers using descriptive statistics and thematic analysis. Results: Twenty-two participants from eight provinces participated in key informant interviews and reported on 17 early cancer diagnosis initiatives. Most initiatives (88%) were in early phases of implementation. Two patient-facing and eight provider/organization barriers to implementation (e.g., lack of stakeholder buy-in and limited resources) and five facilitators for implementation and sustainability were identified. Opportunities to improve early cancer diagnosis initiatives included building relationships with stakeholders, co-creating initiatives, developing initiatives for Indigenous and underserved populations, optimizing efficiency and sustainability, and standardizing metrics to evaluate impact. Conclusion: Early cancer diagnosis initiatives in Canada are in early implementation phases. Lack of stakeholder buy-in and limited resources pose a challenge to sustainability. We present opportunities for funders and policymakers to optimize the use and potential impact of early cancer diagnosis initiatives.

## 1. Introduction

Approximately one in two Canadians will develop cancer in their lifetime and one in four will die from the disease [1,2]. The pre-diagnosis interval, or interval between first patient presentation with symptoms and cancer diagnosis, is a critical part of the cancer care continuum, yet care is often fragmented, characterized by long waits and lack of provider coordination [2,3]. Optimization of this interval, or early cancer detection, can result in improved patient experiences, reduced time to cancer diagnosis, and may contribute to improved effectiveness of treatments and decreased health system costs [1,2,3,4].

The Canadian Partnership Against Cancer (the Partnership) is a federally funded organization that is mandated with effecting systems change to improve cancer control and care for individuals with cancer in Canada. The Partnership aims to reduce cancer incidence, deaths from cancer, and improve quality of life of individuals with cancer. In 2019, the Partnership launched the 2019–2029 Canadian Strategy for Cancer Control [3], which aims to engage actors from all Canadian provinces and territories to optimize cancer care pathways and improve equity of the cancer care system. Specifically, the Partnership identified significant inequities in cancer care for underserved individuals (which can include those who live in rural, northern, and remote Canadian jurisdictions), as well as for First Nations, Inuit and Métis communities [5,6,7,8,9,10,11]. The Strategy outlined eight priorities, one of which is to diagnose cancer faster, accurately, and at an earlier stage. This priority aims to optimize rapid access to diagnosis for individuals with suspected cancer (e.g., individuals who present with symptoms) and to strengthen screening efforts across Canada.

The impact that the COVID-19 pandemic has had on cancer care and patient outcomes is not fully known, however, early studies show significant reductions in access and use of cancer screening and early diagnosis initiatives in Canada [12,13,14,15]. Estimates suggest a 40% decrease in primary care visits, 50% decrease in hospital visits via emergency departments (where ~30% of cancers are diagnosed in Canada), and decreased screening rates across the country [12,13,14,15]. Decreases in primary care visits, hospital visits, and screening rates can potentially impact patient outcomes, as patients are less likely to be diagnosed with early stage cancer unless they are seen by their health care provider for cancer screening or for work-up of new symptoms. As the health care system aims to return to normal, it is imperative that early cancer diagnosis initiatives are optimized to provide care to individuals with suspected cancers. Understanding the barriers and facilitators to implementation and sustainability of early cancer diagnosis initiatives is a necessary first step in this process.

To inform development and operationalization of the Canadian Strategy for Cancer Control, particularly in the current context of the COVID-19 pandemic, the Knowledge Translation (KT) Program, on behalf of the Canadian Institute of Health Research (CIHR) SPOR Evidence Alliance and the Partnership, conducted an exploratory qualitative research study to summarize current perceived barriers and facilitators to implementing and sustaining early diagnosis initiatives in Canada. This study was conducted as the Partnership aims to improve and support the development, implementation, and sustainability of accurate and rapid diagnosis for individuals with symptoms or suspected cancers across the nation; fulfilling the Canadian Strategy for Cancer Control’s priority to diagnose cancer faster, accurately, and at an earlier stage. The objectives of the study were to describe the perceived barriers and facilitators to the implementation and sustainability of current early cancer diagnosis practices across Canada. This information will inform the Partnership’s activities to operationalize their key priority.

## 2. Materials and Methods

### 2.1. Study Design

We conducted an exploratory qualitative research study, composed of key informant interviews and a document review of materials submitted by key informant participants. This study was designed to understand the perceived barriers and facilitators to implementing and sustaining early cancer diagnosis initiatives within the Canadian context including perceived impacts (negative or positive) of the COVID-19 pandemic on early cancer diagnosis efforts. We report our study using the COnsolidated criteria for REporting Qualitative Research (COREQ) [16].

### 2.2. Participants

In collaboration with the Partnership, we purposefully recruited interview participants. Participants were identified via the Partnership’s established networks, which were formed over years of collaboration with healthcare professionals and organizations, patients, and communities [17,18]. These participants were identified by the Partnership as potential study participants if they were directly working on or previously worked on an early cancer diagnosis initiative. The potential list of participants identified initially included a range of initiatives across Canada and in different rural and urban settings. A snowball sampling approach was used to identify additional key informants as needed. Participants were recruited by KT Program researchers via email. In this email, participants were provided the study information sheet. Participants were asked to respond if they were interested and/or if they felt their work fell within the study’s objectives. If participants did not respond, they were sent a follow up email. If they did not reply to the follow up email, they were not contacted again.

### 2.3. Data Collection and Outcomes

After providing informed consent, key informant participants participated in a single interview approximately 60 min long conducted between 28 September 2020–20 January 2021. Interviews were conducted by two experienced researchers (LDH, TL); the interviewers did not hold relationships with the study participants. Interviews were offered in French and English. An interview guide was co-created with the Partnership to identify characteristics of initiatives, whether and how the initiatives were evaluated, and barriers and facilitators to implementation and sustainability of the initiatives. Participants were also asked to describe opportunities to optimize early cancer diagnosis initiatives in Canada and to share relevant documentation (e.g., internal reports, standard operating procedures, webpages, or published articles, triage forms) for described initiatives. In addition, interview participants were asked to provide clarification of any relevant data from the document review. Interviews were audio-recorded and transcribed verbatim. Participants were also followed up to provide documentation related to their work in early cancer diagnosis after the interview if they had not done so prior.

### 2.4. Data Analysis

Participant and initiative characteristics were descriptively analyzed. Where relevant, data derived from the document review were included in these descriptive summaries. Two researchers analyzed the data from the document review and 20% of the documents were double coded until 75% agreement was reached. Following this, documents were reviewed by one researcher and descriptive data were abstracted to a tracking table. These data were used to describe initiatives. Interview transcripts were double-coded by two researchers (LDH, TL) using NVivo 12 qualitative software [19] and analyzed using a thematic analysis approach [20,21] to identify emergent themes. Researchers double coded 20% of the data until 75% agreement was reached; discrepancies were resolved by a third party (CF). In order to review accuracy and complete missing details, study participants were asked to perform a member check [22] on a summary of interview notes and data abstracted from the submitted documents. This study was exempted from research ethics review.

## 3. Results

### 3.1. Participant Characteristics

A total of 22 individuals participated in the interviews. Participant roles varied and included primary care physicians, surgeons, oncologists, and hospital and government administrators. Participants represented eight provinces (*n* = 9 Ontario, *n* = 3 Quebec, *n* = 3 Nova Scotia, *n* = 3 Alberta, and *n* = 1 British Columbia, Saskatchewan, Manitoba, Newfoundland and Labrador, respectively). Fifteen participants contributed to the document review and 19 participants completed the member check.

### 3.2. Initiative Characteristics

Participants described 17 initiatives across Canada. See Table 1 for a summary of initiative characteristics. The initiatives had various (and sometimes multiple) points of entry to the diagnosis program, such as via primary care providers, screening, and/or emergency rooms. Targeted disease types varied by initiative with some providing diagnostic services on more than one disease type. Of the 17 initiatives, 11 were symptoms focused. The remaining initiatives focused on optimizing provider processes, such as primary care provider education (e.g., seminars about how to recognize early cancer diagnosis symptoms in primary care), or standardization of a surgical triage system. The two initiatives focused on care for Indigenous populations provided cultural competency training to staff and developed educational materials to improve patient reach. These initiatives also partnered with Indigenous leaders, Elders or groups in processes of planning, implementation and decision-making. Nearly all (15/17) initiatives were in a planning or early implementation phase and half (8/17) prepared plans for initiative sustainability/spread, although none had begun this sustainability work at the time of interview.

### 3.3. Evaluations of Early Diagnosis Initiatives

Most of the initiatives were in early implementation phases and were beginning data collection; one initiative had completed assessment of intervention impact. Example performance metrics collected included wait times to diagnosis or treatment (with comparisons to provincial wait time targets), wait times from initial presentation to care provider to diagnostic imaging, time from biopsy to report/diagnosis, patient volume and testing, patient satisfaction, and system efficiencies (e.g., repeat tests; percent of emergency department visits leading to diagnosis).

Participants noted that evaluation data on early diagnosis initiatives had the potential to benefit patients, providers and the system by demonstrating weaknesses, strengths, and value; however, resource and personnel shortages were a barrier to routine data collection. The use of digital or virtual platforms was a facilitator to conducting routine initiative evaluations.

#### 3.3.1. Barriers to Initiative Implementation and/or Sustainability

Common barriers and facilitators to implementation and sustainability were identified across the initiatives. We identified ten barriers to implementing or sustaining early diagnosis initiatives in Canada; these are described in detail alongside participant quotes in Table 2. Two barriers were patient facing and eight were provider/organization facing. Patient-facing barriers included lack of access to primary care providers to facilitate referrals/enrollment to initiatives and lack of access to initiatives due to patient geography. Provider and organizational barriers included lack of cooperation from colleagues to participate in initiatives, lack of government/policymaker buy-in, limited staff capacity to support/sustain the initiative, lack of awareness about initiatives and/or screening/diagnosis guidelines (which was closely tied to non-adherence to screening or diagnosis guidelines resulting in delayed access to initiatives), burden on primary care providers to navigate care pathways, lack of data to facilitate reporting of initiative impacts, technological gaps, and limited funding/resources to implement and sustain initiatives.

#### 3.3.2. Facilitators to Initiative Implementation and/or Sustainability

Five facilitators to initiative implementation and sustainability were identified (Table 3). Many of these were the converse of identified barriers and included: leadership and organizational buy-in, data availability on initiative processes and impact, leveraging networks to maintain coordination among stakeholders, small-sized organizing groups, and use of virtual elements.

#### 3.3.3. Opportunities for Early Cancer Diagnosis Initiative Programs and Research

Participants identified strengths and opportunities across Canadian early cancer diagnosis programs. Participants perceived the use of initiatives that included multidisciplinary teams, patient navigators, and central referral systems to contribute to the success of an initiative. Multidisciplinary teams included collaborations among different roles such as, diagnostic imaging, pathology, medical and/or surgical oncology and administration to facilitate cancer diagnosis. Patient navigators acted as a point-person for individuals and staff involved in the early cancer diagnosis pathway. These navigators supported scheduling, communication with patients and coordination with cancer care providers. Many participants described the role of navigators as a “key facilitator” to initiative success and sustainability; participants perceived the use of navigators to be favorable among both patients and providers. Finally, participants perceived use of central referral systems to facilitate use of early diagnosis initiatives. For instance, pathways that provided patients with a single location to complete all diagnosis work-up (e.g., ‘one-stop-shops’) were perceived to improve efficiency and reduce burden on both patients and providers and simplified processes of scheduling, referrals, and testing.

Participants also identified a number of opportunities for program developers seeking to improve or sustain early cancer diagnosis initiatives in Canada. First, participants recommended that initiative developers build relationships with relevant stakeholders, particularly at the policymaker level. Such partnerships between organizations rather than individual relationships between persons (e.g., program developer and policymaker) were perceived to support sustainability of initiatives despite staff turnover. Participants recommended that initiative leads use impact data to gain buy-in from policy/government organizations and to specifically highlight impact on patient or system outcomes. Second, participants highlighted the need to develop early cancer diagnosis initiatives focused on First Nations, Inuit and Métis and underserved populations. Participants recommended developing longstanding collaborations with elders and community leaders and including provider education on culturally safe care approaches. Third, participants stressed the importance of co-creating early diagnosis initiatives with patients, primary care providers, cancer care specialists and administrators. For instance, some participants stressed the importance of including patient advisors on steering committee panels to support and inform initiative development or sustainability; these advisors included individuals with lived experience who are engaged from the onset of initiative development and support initiative processes (e.g., committee meetings) and deliverables (e.g., educational materials). Additionally, co-creation with primary care providers (who are often not engaged in initiative development) may result in streamlined processes for referrals and can support the development of efficient and feasible diagnosis pathways for patients. Fourth, there were perceived opportunities to invest in resources to support initiative efficiency, such as electronic patient records compatible with current systems and across organizations, central referral systems, and diagnosis equipment (particularly in rural areas). Finally, participants reported a need to define key metrics to evaluate initiative success and recommended the stipulation of minimum data collection requirements across initiatives. These data were perceived to be facilitators to policymaker buy-in, thereby improving resource allocation and initiative sustainability. However, participants also highlighted the need for dedicated financial and personnel resources to support such evaluations.

## 4. Discussion

We conducted an exploratory qualitative study composed of key informant interviews and a document review. The study included 22 participants representing 17 early cancer diagnosis initiatives across 8 Canadian provinces.

Our study fills a gap in the literature on systematic exploration of barriers and facilitators to implementing or sustaining early cancer diagnosis initiatives in the Canadian context. The few studies describing barriers to early cancer diagnosis services focused on barriers to accessing existing programs. For instance, a recent systematic review described barriers to accessing lung cancer diagnosis programs [23]. The studies in this review showed that poor relationships between primary care providers and patients, lack of accessibility of services for patients due to geography, and patient and provider lack of awareness of cancer symptoms and treatments were barriers to access. Additionally, these barriers are compounded for underserved or underrepresented communities, racial minorities, immigrant populations, or those living in rural or remote areas [24,25]. Our study identified 10 barriers at the patient, provider and organizational levels that challenge initiative implementation and sustainability and five facilitators that can be leveraged to optimize initiative delivery.

Participants in our study identified opportunities to address gaps in early cancer diagnosis care in Canada. Indigenous peoples’ cancer rates continue to rise, yet screening rates are lower compared to non-Indigenous populations [26]. This presents an opportunity to standardize the use of cultural competency programs across early cancer diagnosis initiatives and to co-develop tailored pathways or services to support Indigenous individuals presenting with suspected cancer. Similar opportunities exist to provide tailored initiatives and improve accessibility for other priority groups, such as immigrant or non-English speaking populations and individuals living in rural areas.

There is a critical need to co-create diagnostic initiatives with patients and primary care providers. A recent systematic review failed to identify any interventions that involved patients in the process of early cancer diagnosis after initially presenting to primary care [27]. Additionally, this review identified many barriers at the primary care provider level; mainly, these were attributed to primary care providers being over-burdened and feeling unsupported to keep up with multiple referral systems or rapidly changing diagnosis guidelines. Co-creation of streamlined pathways with primary care providers may improve feasibility and efficiency of initiatives and may result in improved buy-in and uptake of programs by both primary care physicians and cancer care providers [28].

Finally, our study revealed that budget cuts often preclude initiative administrators from collecting evaluation metrics; those that conduct evaluations vary significantly with regards to the type and frequency of data collected. These challenges have been indicated in the literature [29,30]. Tools such as the Aarhus checklist, developed using a systematic review of instruments to measure outcomes in early cancer diagnosis research and an expert consensus approach, provide guidance on minimum reporting criteria of early cancer diagnosis initiatives [31]. Consistent reporting using such templates and standardized evaluation metrics can promote consistency and transparency in early diagnosis definitions and may facilitate process (e.g., wait times, time to diagnosis) and impact (e.g., survival, treatment completion) comparisons across interventions, regions or organizations. Given many of the early diagnosis initiatives in Canada are in early implementation phases, there is a timely opportunity to standardize routine data collection to assess process and impact outcomes.

Our study is not without limitations. Our interviews were limited to 22 pan-Canadian participants and participant representation from the territories and two Atlantic provinces were lacking. Therefore, data included in this report and the 17 initiatives highlighted are not representative of all existing early cancer diagnosis initiatives in the country and may have overlooked other efforts. This study was not intended to be a comprehensive assessment of initiatives and instead the focus was to understand barriers and facilitators to implementing early cancer diagnosis initiatives. There was no specific framework that guided the analysis since this was an exploratory study. Next steps for consideration can include using the Theoretical Domains Framework to categorize the barriers and facilitators, which can inform development of strategies to overcome the barriers and leverage the facilitators [32]. However, our findings are consistent with a previous study conducted in 2018 [6], which identified similar challenges and opportunities to advance early diagnosis cancer care in Canada, suggesting there has been little progress made with respect to implementation of early diagnosis initiatives in recent years. Finally, our data after reaching a 75% agreement by two researchers on the first 20%, were only reviewed independently and therefore data may have been missed or interpreted from one perspective; however, this risk was mitigated through participant validation, or member checking of data.

Diagnosing cancer faster, accurately and at an earlier stage is a key priority of the 2019–2029 Canadian Strategy for Cancer Control [3]. Over the next five years, the Canadian Partnership Against Cancer will leverage findings from this exploratory qualitative study, as one of several inputs, and partner with Canadian jurisdictions to continue to test innovative models of care that expedite cancer diagnosis, especially for First Nation, Inuit, and Metis peoples and underserved populations.

## 5. Conclusions

Cancer diagnosis initiatives in Canada are in early implementation phases; stakeholder buy in and limited resources challenge the sustainability of these interventions. In this paper, we present ten barriers and five facilitators to implementing and sustaining early cancer diagnosis initiatives, along with key opportunities for funders and policymakers to optimize the use and potential impact of such initiatives.

## Figures and Tables

**Table 1:** Initiative characteristics.

Initiative Characteristics		Number of Initiatives (*n* = 17)
Size		
	National	1
	Provincial	8
	Regional/Local	8
Point of Entry ^1^		
	Primary care provider/usual care	12
	Screening	3
	Patient navigator	2
	Hospital specialist referral	6
	Emergency room	2
	Walk-in/Urgent care clinic	2
Disease Type ^1^		
	Breast	3
	Melanoma	1
	Endometrial	1
	Ovarian	1
	Thoracic	3
	Pancreatic	1
	Colorectal	1
	All-cancers	4
Initiative Focus		
	Symptoms	11
	Optimizing provider processes (e.g., primary care provider education, standardizing surgical triage system)	6
Underserved and/or Indigenous focused care		
	Yes	8
	No	9
Digital and/or Virtual Elements Included (e.g., online standardized referral form)		
	Yes	9
	No	8
Collecting Evaluation Metrics		
	Yes	13
	No	4

^1^ Initiatives may have had more than one point of entry and thus N will equal more than 17 initiatives. For example, a single initiative may have had 3 different points of entry.

**Table 2:** Participant quotes demonstrating barriers to initiative implementation and/or sustainability.

Barrier	Description	Example Quote(s)	Implementation/Sustainability Barrier	Patient/ Provider/ System Barrier
Lack of access to primary care providers	Patients lack access to primary care physicians. These patients typically enter the system via emergency rooms or walk-in clinics, which may delay time to diagnosis. Lack of primary care access is exacerbated for underserved communities and individuals with limited health literacy.	“The family medicine access here is poor…I believe probably half those patients are entering through the emergency room.”—Oncologist “Of course, there’s a population who don’t have a regular family doctor. And so they might enter through a walk-in clinic type thing. Urgent care, sometimes emergency department if their symptoms are getting more severe.”—Senior Administrator	Implementation	Patient/System
Lack of access to early diagnostic programs due to geography	Patients in rural communities are required to travel further (often to urban areas) to access early cancer diagnostic programs or to receive a cancer diagnosis. This was specifically highlighted among individuals with lung cancer.	“With the big challenge for us, though, also is geography. We serve about two million people in [location]. It’s quite spread out right, as people who will come and travel five or six hours to see us. That’s a big commitment, right, for them.”—Surgeon “From most communities, you take at least two planes to get to [central city]. So it’s a huge other load of issues to be concerned about over your health issues.”—Senior Administrator	Implementation	Patient/System
Lack of cooperation from colleagues	Practitioners may have limited buy-in (e.g., unwillingness to use early cancer diagnostic pathways, guidelines). This was pronounced when initiatives impact perceptions of existing hierarchies/roles (e.g., use of multidisciplinary clinics). Additional barriers include lack of cooperation between multiple organizations and lack of buy-in among an organization’s administration.	“And that’s the biggest thing I’ve encountered in terms of learning how to navigate this bureaucracy where everybody’s trying to protect their own little silo or whatever. Instead of trying to work together”—Surgeon “So I think the most important thing is that it can be challenging to get people wearing a lot of different hats to trust each other and come together with a common agenda. And that takes a lot of work.”—Medical Director	Implementation/Sustainability	Provider
Lack of government/ policymaker buy-in	Often, government buy-in is associated with funding, resources, oversight or guidance; without this buy-in, initiative leaders are required to secure these resources and collaborations independently.	“I think the geopolitical climate can be a barrier depending on what’s going on. And as you know, in [province] right now, there’s some sticky issues. “one of our biggest barriers when we come up with when things are developed or when you’re trying to get a program like a new screening program going, you have to have buy in at the Ministry of Health because they’re the funder. And without the money to fund it, it just isn’t going to happen.”—Medical Director	Sustainability	System
Limited staff capacity to support/sustain initiative	Early cancer diagnostic initiatives often require significant administrative efforts to coordinate and sustain. These tasks are compiled to busy providers’ tasks which adds increased burden and decreases motivation for providers to participate in the initiative.	“I think the big one for the navigators can they’ve gotten really busy, which is fantastic. You know, they’re really busy because there’s no resources like they’re doing a lot of clerical stuff and that remains a barrier. So they spend a lot of time faxing and know entering data and typing and computers and that kind of stuff. And that’s not really the best use of their time.”—*Oncologist* “And it was exceptionally frustrating as a family physician because you literally spent hours banging your head against the wall, doing personal emails to everybody under the sun to try to get somebody to care for your patient. And it’s that frustrating for me. Imagine it’s like for the patient right now. It’s certainly unacceptable.”—Medical Director	Implementation/Sustainability	Provider/System
Lack of awareness about initiatives or guidelines	Among providers, particularly primary care providers, there was lack of awareness on how to use or access early diagnostic initiatives, particularly new diagnostic pathways/guidelines.	“And it was exceptionally frustrating as a family physician because you literally spent hours banging your head against the wall, doing personal emails to everybody under the sun to try to get somebody to care for your patient. And it’s that frustrating for me. Imagine it’s like for the patient right now. It’s certainly unacceptable.”—Medical Director “One thing I would say on that that’s exceptionally important, which people don’t realize the importance of, if a rapid diagnostic clinic wants to be accessible, do not throw barriers up to family doctors. What they all tend to do is they say, “I have my own referral form”… So what I say to all of the sites is you must, must, must if you want referrals, you must accept our referral and whatever form we choose to send it to you. Right… Please be open to other means. Make it easy for the referring family physician, because we spend a lot of hours trying to connect with people to get our patients that really need help.”—Medical Director “There are gaps for patients. They are faced with delays that create high levels of anxiety and distress. From the primary care provider perspective, there is no organized and coordinated intake process for patients with suspicious cancer symptoms or signs. It’s on the backs of family doctors to figure out how to get a positive diagnosis and then specialty programs (cancer centres) will then accept referrals for those patients. There’s evidence and data that delays not only impact patient anxiety, but can impact disease severity,”—Senior Administrator	Implementation	Patient/Provider
Non-adherence to screening/diagnostic guidelines	Providers perceived these guidelines to change frequently and also perceived guidelines to have different thresholds for decision making (e.g., when a test should be ordered) which leads to inconsistent care across providers. Primary care practitioners felt it was their responsibility to remain up-to-date on changing guidelines, which was challenging given already busy schedules.	“Since I know the breast world, if you look at, women who have a symptom and they say, “well, I’m 30, so I don’t need a mammogram, because I heard that women under the age of 40 don’t need a mammogram”. They’re [the women] not sophisticated to enough to know between diagnostic and screening. And then all of this data that comes out that mammograms are over calling unnecessary and choosing wisely. And, we [the family physicians] have to really think about the impact that has on the frontline women and engage them in that conversation, because we’re [family physicians] not doing a great job of that right now.”—Medical Director “People were waiting too long or having inappropriate tests done, you know, with no kind of guidance for the patients from health professionals….We’re putting out a lot of excess tests that aren’t necessary. Family doctors don’t know that’s the problem. And family doctors still think for the most part that [disease type] is incurable. Right. This is a very, very commonly held view in Canada.”—Surgeon “Stakeholder engagement is important. When you think that you’ve communicated enough, communicate again. It can be challenging to navigate changes [in a system].”—Program Manager	Implementation	Provider
Burden on primary care providers	Primary care practitioners expressed frustration regarding the added burden on primary care practitioners to use early cancer diagnostic initiatives (e.g., completing several referral forms for patients, administrative tasks to ensure patient is referred appropriately).	“And the from the primary care provider perspective, there is no organized and coordinated intake process for suspicious patients with suspicious cancer symptoms or signs. It’s on the backs of family doctors to figure out how to get a positive diagnosis.”—Senior Project Manager	Implementation/Sustainability	Provider/System
Lack of data to facilitate reporting of initiative impacts	Limited resources preclude administrators from routinely collecting initiative impact data. Budget cuts to initiatives often force administrators to sustain clinical work at the expense of ongoing data collection. These lack of data then pose a challenge to initiative sustainability, as policymakers require this impact data to make decisions for ongoing funding.	“Even to get going, we need background data that helps us secure funding for the projects identify. You know, the problem kind of defines the problem attention”…[Data can] get you off the ground…with funding. [Data will] get you more funding and more buy in with the return on investment argument.”—Senior Project Manager	Sustainability	Provider/System
Limited funding/ resources	Limited funding is a barrier to both the expansion and sustainability of early diagnostic initiatives. Participants perceived the COVID-19 pandemic as a challenge to early cancer diagnostic funding. Additionally, lack of necessary equipment or physical resources (e.g., CT or MRI) was a barrier to implementing initiatives; this was a challenge observed in many rural regions.	“We don’t even have a scan or MRI or any of those, diagnostic equipment up north. We can do some X-rays. We can do certain basic lab tests. But anything that goes beyond in terms of investigation, we would have to send the person to [central city] for further testing.”—Planning and Programming Officer “So I hate to reduce this to one issue, but funding constraints are probably the major impediment to that.”—Medical Lead	Implementation/Sustainability	System
Technological gaps impact initiative efficiency	Fax machine delays, lack of EMR accessibility and image retrieval software impacted the efficiency of early cancer diagnostic initiatives.	“The bottleneck in our system right now is the papers get handed around and it takes a long time from the time a family doctor sends it in and it sits on a fax machine, goes to the guy, the guy looks at it, the guy sends it back and the next guy looks at it. So the data we’ve tracked recently, that takes five to seven days just to get the paper to the person that’s going to do the tests.”—Surgeon “They’re not always able to pick up images electronically and view them so that your radiologist can do, for example, the image guided biopsy. And so then it’s repeat or slightly archaic, but it actually happens…Patients are asked to bring CDs of their mammograms or their images from one spot to another. So, as you can imagine, there’s a lot of issues with that.”—Group Manager	Implementation/Sustainability	System

**Table 3:** Participant quotes demonstrating facilitators to initiative implementation and/or sustainability.

Facilitator	Description	Quote	Implementation/Sustainability Facilitator	Patient/Provider/System Facilitator
Facilitator to initiative implementation/sustainability				
Leadership and organizational buy-in	Engaging organizational (e.g., department chairs) and government (e.g., ministries of health) leadership facilitates increased stakeholder awareness of initiative, coordination among sites (thereby facilitating scale up), and improved resource allocation. This was particularly noted for provincial-level initiatives that require multi-level organizational buy-in.	“It was an institutional project. The administration was behind us and made it a priority. The project needed that. It took the administration supporting us to do this project.”—Program Director “We have the strength of the entire [provincial organization] system as well …once we put the [provincial organization] label on it, then it’s a very effective action moving forward, especially if we also have support of surgical oncology.”—Program Manager	Implementation/Sustainability	Provider/System
Data availability on initiative processes and impact	Impact data on initiative success (e.g., who the initiative served; impact on patient-important and clinical outcomes) was of value to both internal and external initiative stakeholders and facilitated buy-in. These data can also be used to iteratively make improvements to initiative processes and reach.	“I think it’s just capturing all those wait times. So we were able to show to go back and look. Now we’ll be able to show that we sort of cut the wait time to get to a transition by, I think more than a half. Like more than 50 percent.”—Oncologist “And so having the data to say, OK, here’s what we’re seeing, does this resonate with you? What does this look like? OK. There’s an issue here. What are what are some of the strategies having that foundation and data is a huge enabler. And at the same time, it’s very difficult to have exactly the data you need at the granular level that you need as well. So it can be a bit of a challenge. But where we have it, we leverage it and it’s very effective.”—Program Manager	Implementation/Sustainability	Provider/System
Leveraging networks to maintain coordination among stakeholders	A network of colleagues working together towards a shared goal was essential to expediting diagnostic processes and sustaining early cancer diagnostic initiatives. These networks were particularly useful to facilitate collaboration across clinical departments or specialties.	“[We have] got the advantage of being a…clinical network…We’ve got that relationship with…15 others besides us and so we can draw them in and work very collaboratively as needed. They’ve got very broad networks as well. So we can leverage that out as required to help with the work that we’re doing”—Senior Administrator	Implementation/Sustainability	Provider/System
Smaller sized organizing groups	Some participants reported the utility of an ‘implementation team’ responsible for day-to-day initiative processes. Smaller teams were also perceived to facilitate more streamlined discussion of patient cases.	“The smallness, in that we there’s a small number of people that we can communicate pretty easily. It wasn’t too complicated to do. It wasn’t like we had multiple centers that join together and pull this off.”—Oncologist “And also because it’s going to the three of us, we’ll discuss the cases every Monday together as a group with input from pathology, radiology, gastroenterology…Things like that. These are all discussed and we can get things going quickly so that when we send the consult to the medical oncologist, to the radiation oncologist, they already know about it because we’ve already discussed it.”—Surgical Lead	Implementation	Provider
Use of virtual elements to facilitate care	Virtual platforms to enhance patient population reach (particularly for those living in rural areas), promote patient and provider education, and support initiative efficiency (e.g., EMR capabilities) were identified as a facilitator to implementation and sustainability.	“We’re using virtually a lot at our institution, both for educational, for all of our meetings…patient engagement and support. We have some support groups [for patients]”—Medical Director “I can say in primary care, 80 percent virtual, 20 percent in person…[and] there’s every intention that virtual will persist beyond the pandemic and will be utilized more”—Medical Director	Implementation/Sustainability	Provider/System

## Data Availability

Data sharing is not available for this article due to the confidentiality and privacy assured to the participants.
